# A comparison of cardiovascular imaging practices in Africa, North America, and Europe: two faces of the same coin

**DOI:** 10.1093/ehjimp/qyad005

**Published:** 2023-07-10

**Authors:** Suvasini Lakshmanan, Irina Mbanze

**Affiliations:** University of Iowa Hospitals and Clinics, Iowa City, IA; Cardiology Service, Department of Medicine, Maputo Central Hospital, 1653 Avenida Eduardo Mondlane, Maputo, Mozambique

**Keywords:** cardiovascular imaging, Africa, North America, Europe, burden of CV disease

## Abstract

Cardiovascular diseases remain the leading cause of morbidity and mortality worldwide. There are significant differences in the burden of cardiovascular disease and associated risk factors, across high-income countries and low- and middle-income countries. Cardiac imaging by echocardiography, cardiac computed tomography, cardiac magnetic resonance imaging, single-photon emission computed tomography, and positron emission tomography myocardial perfusion imaging are well-established non-invasive tests that aid in the diagnosis, risk stratification, and management of various cardiac diseases. However, there are significant inequalities in availability and access to imaging modalities in low- and middle-income countries attributed to financial constraints, disparities in healthcare and technical infrastructure. In the post-COVID-19 pandemic era, these disparities are exaggerated by the continued technological advancements driving innovations in the field of cardiovascular (CV) imaging in high-income countries, while there is an urgent need to provide sustainable access to diagnostic imaging for patients in economically strained healthcare systems in regions like Africa. This review aims to highlight the inequalities in the burden of cardiac disease, associated risk factors, and access to diagnostic CV imaging tests, while also exploring the need for sustainable solutions to implementing CV imaging all over the world.

## Graphical Abstract

**Graphical Abstract:**
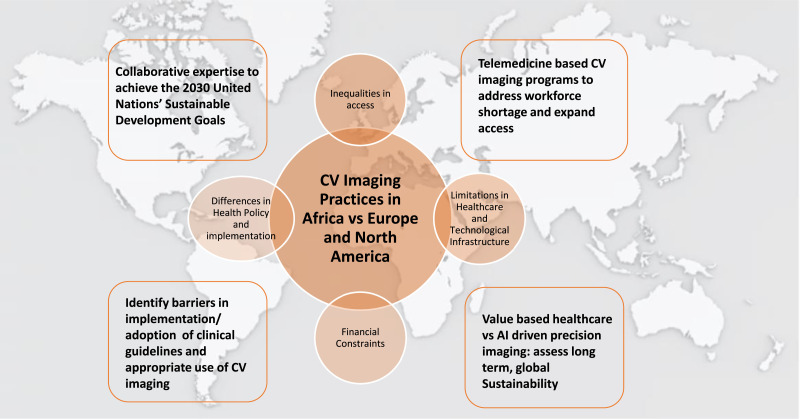
Cardiovascular imaging practices in Africa vs. North America/Europe: challenges and need for global sustainability.

## Introduction

Cardiovascular disease (CVD) is a growing public health concern globally, with an increase in incidence and associated morbidity, mortality, and economic burden. In the last two decades, there have been significant declines in age-adjusted prevalence of coronary heart disease, stroke, and associated mortality and disability-adjusted life years across the world, but disparities in burden of CVD between high-income countries (HICs) and low- and middle-income countries (LMICs) persist, highlighting the urgent need to address the implementation of global policies to promote accurate diagnosis, monitor progress, and accurately guide preventative measures and interventions.^1^ The Global Burden of Cardiovascular Diseases collaboration has sought to characterize the distinct global epidemiologic landscape of burden of CVD and attributable modifiable and non-modifiable risk factors identified by various socioeconomic demographics and geographical regions in the world.^2^ Real world data continues to particularly highlight the differences in burden of CVD, associated risk factors, structural heart disease, and pervasive disparities in diagnosis and preventive care, between various regions of Africa and the high-income regions of Europe and North America.^3^ Multimodality cardiac imaging is growing to be the cornerstone in diagnosis, risk stratification, and management of patients with complex pathology of structure, function, and vasculature of the heart.^4^ In the background of tremendous technological and therapeutic advancements in cardiovascular imaging and care in HICs, there is significant global inequality in availability and access to diagnostic cardiac imaging tests in LMICs attributed to differences in healthcare personnel and technical infrastructures, financial costs, and technological limitations, across HICs and LMICs. In this brief review, we explore, compare, and provide an overview of current state of prevalence of heart disease, risk factors, and differences in access to CV imaging across North America, Europe, and Africa. Understanding these differences is crucial for developing effective prevention and treatment strategies tailored to each region’s unique needs, and reinforces the necessity of a unified, sustainable vision for leveraging technological advancements in CV imaging to bridge the divide in access and cardiovascular care globally.

## Landscape of burden of cardiac disease

The Global Burden of Disease (GBD) Study in 2021 compiled a summary of disease estimates across Africa, Europe, and North America, which affords us to identify region-specific burden of disease and risk factors, which can guide our policies for risk stratification and management. Globally, the age-adjusted prevalence of total CVD was 7241.7 per 100 000 and disability-adjusted life years was 4942.3. Predominantly similar trends were observed in North American and Western Europe with the age-adjusted prevalence of total CVD at 7843 per 100 000 and 6907.5 per 100,000, respectively. Significantly contrasting data with high prevalence of heart disease were observed in various regions of Africa with an age-adjusted prevalence of 9978.7 per 100 000 in North Africa, 8091 per 100 000 in western sub-Saharan Africa (SSA), and 7691.9 per 100 000 in central SSA.^5^ These disparate distribution of heart disease and risk factors contribute to differential cardiovascular outcomes across the world.

CVD remains a major public health challenge in Africa, with variations in prevalence observed between different regions of the continent. According to the most recent data from the World Health Organization (WHO), the prevalence of CVD in SSA has increased by 28% over the past 25 years, with hypertension (HTN) being the leading cause of CVD-related morbidity and mortality in the region.^6^ The Sub-Saharan Africa Survey of Heart Disease Registry is a large-scale study that aimed to provide insight into the prevalence, incidence, and outcomes of CVD in SSA. The study collected data from 12 African countries and found that CVD is a major and growing public health concern in the region. The study revealed that HTN is the most common risk factor for CVD in SSA, affecting over 30% of the population. Similarly, the INTERHEART Africa study identified HTN, smoking, diabetes, hyperlipidaemia, and obesity as main risk factors that contributed to nearly 90% of acute myocardial infarction, in SSA.^7^ The study also revealed that the incidence of heart failure in Africa is increasing, with high mortality rates reported. The cause of heart failure remains predominantly non-ischaemic, with rheumatic heart disease and endemic cardiomyopathies (idiopathic dilated cardiomyopathies, peripartum cardiomyopathies, and endomyocardial fibrosis) accounting for 75.5% of the cases, although the rate of ischaemic heart disease may have been underestimated owing to limited diagnostic tools.^8^ In East Africa, rheumatic heart disease continues to be a major public health problem, with a prevalence of up to 10% in some regions attributed to poor living conditions, inadequate medical care, and poor nutrition.^9^ In West Africa, there is a high prevalence of heart failure, with HTN, diabetes, and obesity being the leading risk factors.

In the 2023 update of Heart Disease and Stroke Statistics in the USA from the American Heart Association, the prevalence of CVD comprising of heart disease, heart failure, stroke, and HTN in adults ≥20 years of age was 48.6% overall (127.9 million in 2020) and shown to increase with age in men and women.^10^ The poor cardiovascular health in the USA is associated with high age-adjusted prevalence of HTN and increasing incidence of obesity and metabolic syndrome in adults and US youths. In addition, heart failure is a growing public health and economic burden in the USA, with an estimated incidence of ∼1 000 000 new patients with heart failure per year, while valvular heart disease predominantly affects the elderly over 80 years of age and older, with a general prevalence of 1.5 million people with aortic stenosis, and an estimated 4 million people with significant mitral valve insufficiency.^11–13^

## Challenges in cardiac imaging

Non-invasive diagnostic imaging modalities such as echocardiography, cardiac computed tomography (CT), nuclear imaging, and cardiac magnetic resonance imaging (MRI) play a key role in diagnosis, risk stratification and therapeutic decision-making of heart disease, and influencing outcomes. Access to appropriate, innovative cardiac testing is important to curb the disparities in burden of cardiac disease and outcomes, particularly in Africa and marginalized populations in HICs in North America. Currently, we are at the crossroads of accelerated innovations in cardiovascular imaging with newer technologies, applications of artificial intelligence and explosive utilization of tests and imaging biomarkers to guide management of CVD in HICs challenging value-based systems of care, while there are persistent concerns regarding access and limited utilization in economically burdened regions across the globe.^14^

As discussed above, Africa represents one of the most populated regions with significant burden of cardiac disease and risk factors, further confounded by limited access to healthcare providers, diagnostic imaging, and financially strained health systems. According to the latest data from the WHO, some African countries, such as South Africa, Egypt, and Morocco, have a relatively high number of imaging devices per capita, including echocardiography, cardiac magnetic resonance (CMR), CT, and single-photon emission computed tomography (SPECT)-myocardial perfusion imaging. However, many countries, particularly those in SSA, have limited access to these technologies, with fewer than one imaging device per million individuals in some countries, such as Mali, Niger, and Chad. For example, SSA has an average of 0.3 MRI units per million individuals, with 11 countries with populations ranging from 0.7 million to 67.5 million having no scanners at all. Only eight countries provide CMR services on the African continent, with most scanners concentrated in South Africa and limited to the private sector and academic centres.^15^ This situation is dramatic if we compare the situation in Europe and North America which have a high density of MRI units at 22.2 per million population. The high acquisition and ongoing maintenance costs of CMR pose significant obstacles to adoption, which is further compounded by healthcare worker shortages and limited technical expertise, such as engineers and medical physicists to service and repair such devices. When examining the epidemiology of CVD and testing patterns in Mozambique, CV care is limited by access to only 30 cardiologists currently vs. 12 cardiologists in 2012, for a population of ∼30 million inhabitants. Rheumatic heart disease, hypertensive heart disease, peripartum, and dilated cardiomyopathy are a few of the common conditions; however, access to echocardiography is limited to specialty referral centres with two machines for the southern region, one for the central and one for the northern regions of the country.^16^ To the best of our knowledge, there are no CCTA, cardiac SPECT/positron emission tomography (PET) cameras, or cardiac MRI in Mozambique. These data continue to highlight the gross inequalities in access to basic cardiovascular testing and care in certain low-income countries. There is limited data regarding the use of other modalities such as cardiac CT and access to innovative applications in echocardiography such as 3D echo in other parts of Africa, however, given common underlying concerns regarding limited access to healthcare and technology the concerns for limited utilization of all imaging modalities remain.

The disparities in the use of different types of diagnostic tests and volumes of diagnostic services have been further exaggerated by the COVID-19 pandemic, where the International Atomic Energy Agency conducted a worldwide survey from 669 centres in over 100 countries, and reported significant reductions in diagnostic procedure volumes worldwide of 64% during the peak waves of the pandemic from March 2019 to April 2020, with significant recovery to pre-pandemic levels in HICs and upper middle-income countries (recovery rates of 108 and 99%) in North America, Europe but the recovery rates in low-income countries in Africa remained less robust at 30%.^17^ These findings could predict staggering collateral impact on the CV outcomes, since the data suggest that baseline utilization pre pandemic in March 2019 was up to five times more in HICs in North America and Europe, compared with Africa despite the higher prevalence of cardiac disease.

These challenges are further compounded by limitations in the adoption of clinical guideline recommendations and appropriate use of CV imaging, across HICs and LMICs. The EURECA registry, a prospective international multicentre registry evaluated the 2019 European Society of Cardiology Guidelines based use of cardiac imaging for chronic coronary syndromes, across European, North African (Egypt), and non-European (Brazil, Singapore) countries. Despite adequate access to imaging centres, the utilization rates of imaging tests as recommended in guidelines in patients with chronic coronary syndrome were relatively low.^18^ These results highlight the need to identify barriers to adoption of cardiac imaging in evaluation of CVD, beyond access/availability and may include lack of awareness, financial constraints and health policy.^19^

## Need for global sustainability in CV imaging

As described above, there are increasing concerns to suggest the widening disparities in burden of cardiac disease and risk factors, further exaggerated by global inequalities in availability and access to cardiac imaging as diagnostic tests in evaluation and management of cardiac disease, in developing and developed countries. There is an urgent need to explore the key barriers, and develop sustainable, unifying solutions to implementing cardiovascular imaging namely echocardiography, cardiac CT, cardiac MRI, SPECT, and PET myocardial perfusion imaging in an equitable manner in low-income countries and HICs. The economic role of cardiac imaging will probably remain a primary challenge in achieving sustainable and equitable access to advanced cardiac testing, especially in low-income countries. Growth in non-invasive testing has often been linked with escalating costs involved with infrastructure development, technological innovations, and training of expert personnel, resulting in ‘high-cost healthcare’ in developed nations, underlining the need to address socioeconomic sustainability and shifting the focus to value-based care.^14^ It is likely imperative for national and international imaging societies to work with key stakeholders in health policy, advocacy, and innovators in devices and technology, and leverage the collaborative expertise to achieve the 2030 United Nations’ Sustainable Development Goals.^20^ There is emerging data on efficacy and feasibility of implementing portable, low-cost imaging in resource-limited settings. ‘Cardiac ultrasound for resource-limited settings’, a context-specific protocol, was shown to aid in the diagnosis and management of heart failure and cardiomyopathies in resource-limited settings in SSA.^21^ Low-field CMR is being evaluated as an economically viable and suitable alternative to standard CMR in resource and technologically strained healthcare systems globally.^15^ The Risk Underlying Rural Areas Longitudinal Cohort Study will examine implementation of mobile examination units with built in single heart beat cardiac CT scanners for risk stratification by coronary calcium, to inform poor CV outcomes in rural, low-income counties in counties in Alabama, Kentucky, Louisiana, and Mississippi, in the USA.^20^ Telemedicine-based CV imaging programmes to address workforce shortages and expand access to care are being studied for feasibility and cost effectiveness. A telemedicine programme at the Uganda Heart Institute in East Africa developed a framework for remote transmission of echocardiograms and reporting, and was shown to improve diagnosis, increase prescription of medications and was delivered at a low cost of $29.48/visit.^22^ It is imperative that sustainability efforts expand their focus beyond healthcare infrastructure and costs to high-quality, unbiased, systematic education and research, to remain accountable to our patients globally.^23^

As a global community in CV imaging, we need to acknowledge that the scope of artificial intelligence–driven precision medicine in imaging and sustainability towards value-based care for our patients is dependent on each other, and merely represent two faces of the same coin.

## Data Availability

No new data were generated or analysed in support of this research.

## Lead author biography

Irina Mbanze is a cardiologist at Maputo Central Hospital, in Maputo, Mozambique. During her training, she realized that it would be a challenge to be a cardiologist, as a woman living in a developing country with few resources to diagnose and treat patients with CVDs. But her passion and focus on her purpose allowed her to reach her desired degree in March 2019. Since then, she has been actively involved in campaigns to raise awareness of preventable CVDs, and she has participated in the Pan African Registry of Pulmonary Hypertension and an observational study of sleep disorders breathing in patients with acute heart failure. Dr Mbanze received an International Training Scholarship Award from the American Thoracic Society in 2015. This year she was one of 22 young Cardiologists selected to participate in the first African Course of heart failure, organized by PASCAR. She was also one of the five cardiologists selected to present projects at the 15th PASCAR Congress in association with the Kenya Cardiac Society. In 2021, she won the ‘2022 Women as One Escalator Awards Mentor Match Award’ in the mentee category. Dr Mbanze’s research focuses on better understanding of the CVDs that are prevalent in Mozambique. She is a mother of two who tries to do her best to balance work and family.
